# Clinical Characterization of Mismatch Repair Gene-Deficient Metastatic Castration-Resistant Prostate Cancer

**DOI:** 10.3389/fonc.2020.533282

**Published:** 2020-10-07

**Authors:** Senlin Ye, Haohui Wang, Kancheng He, Mou Peng, Yinhuai Wang, Yuanwei Li, Shusuan Jiang, Jin Li, Lu Yi, Rongrong Cui

**Affiliations:** ^1^Department of Urology, The Second Xiangya Hospital of Central South University, Changsha, China; ^2^Department of Urology, Hunan Provincial People’s Hospital, Changsha, China; ^3^Department of Urology, Hunan Cancer Hospital and The Affiliated Cancer Hospital of Xiangya School of Medicine, Central South University, Changsha, China; ^4^Department of Urology, Central Hospital of Xiangtan, Changsha, China; ^5^Department of Metabolism and Endocrinology, Second Xiangya Hospital of Central South University, Changsha, China

**Keywords:** mismatch repair deficiency, castration-resistant prostate cancer, next-generation sequencing, cell-free DNA, novel hormone therapy

## Abstract

Mismatch repair-deficient (dMMR) prostate cancer is rare and has not been well studied. We aimed to evaluate the clinical characterization of dMMR metastatic castration-resistant prostate cancer (mCRPC) patients. The MMR genes include MLH1, MLH3, MSH2, MSH6, PMS1, PMS2, and EPCAM, and were analyzed by targeted sequencing of plasma cell-free DNA samples. A total of 109 mCRPC patients were identified, including 50 patients with MMR alterations (pathogenic alterations, *n* = 7; alterations of unknown significance, *n* = 43) and 59 patients with wild-type MMR. For the seven patients with pathogenic MMR alterations, the median age at diagnosis was 63.5 years, and 42.9% had a Gleason score ≥8. The median time from androgen deprivation therapy (ADT) initiation to CRPC was 24 months. Compared with the wild-type MMR subgroup, patients with MMR alterations, pathogenic MMR alterations, or MMR alterations of unknown significance showed higher rates of hotspot missense mutations or copy number amplifications in the AR gene (24/50 vs. 10/59, *P* = 7.8 × 10^–4^; 7/7 vs. 10/59, *P* = 2.5 × 10^–5^; 17/43 vs. 10/59, *P* = 0.013). The presence of any MMR alterations was associated with an inferior response to abiraterone [median progression-free survival (PFS): 5.0 vs. 10.9 months, *P* = 0.022]. Shorter PFS times were observed in both the pathogenic MMR alteration subgroup (median PFS: 5 months) and the MMR alterations of unknown significance subgroup (median PFS: 5.3 months), compared with the PFS of those with wild-type MMR genes (median PFS: 10.9 months, *P* = 0.052). There was no statistically significant difference in response to docetaxel chemotherapy between the MMR alterations of unknown significance and the wild-type MMR subgroups (median PFS: 8.2 vs. 8.1 months, *P* = 0.23). Our results demonstrate that dMMR mCRPC patients have an equivalent response to standard ADT and taxane-based chemotherapy treatments compared with wild-type MMR patients. Patients with both pathogenic and unknown significance alterations of MMR genes had poorer responses to abiraterone therapy.

## Introduction

The mismatch repair (MMR) system plays a critical role in the overall fidelity of DNA replication. There are seven known MMR genes (MLH1, MLH3, MSH2, MSH6, PMS1, PMS2, and EPCAM) that can recognize and repair single base–base mismatches or defective insertion–deletion loops (1, 2). Loss of function in any MMR gene leads to hypermutation and high microsatellite instability (MSI-H) due to repair mistakes in DNA replication and recombination (1, 3).

The association between MMR deficiency and MSI-H has been intensively studied in hereditary non-polyposis colorectal cancer, also known as Lynch syndrome (4, 5). Patients with Lynch syndrome have an elevated risk of developing multiple cancer types, especially gastrointestinal malignancies and endometrial and ovarian cancers (6, 7). Raymond et al. reported that men with Lynch syndrome also experienced a 30% increase in their risk of developing prostate cancer by 80 years of age (8).

Mutations in MMR genes are rare in prostate cancers, occurring in 2–5% of cancers that are mostly in the MSH2 and MSH6 genes (9, 10). The loss of MMR genesis is associated with an increased mutational load (11) and an elevation in tumor neoantigens (12, 13). Mismatch repair-deficient (dMMR) solid tumors are sensitive to the immune checkpoint inhibitor pembrolizumab (14). While MSI-H/dMMR prostate cancers are reported to benefit from treatment with PD-1 inhibitors (15, 16), the clinical features of dMMR-genotype metastatic castration-resistant prostate cancer (mCRPC), and the responses of this type of cancer to the standard systemic therapies, are still poorly understood.

In this study, we identified 50 mCRPC patients with alterations in MMR genes by using a targeted deep sequencing approach. We aimed to determine the clinical outcomes of the dMMR genotype mCRPCs in response to novel hormone therapies and taxane chemotherapy.

## Materials and Methods

### Patient and Study Design

From December 2017 to July 2019, next-generation sequencing was performed on consecutive cases of mCRPC from The Second Xiangya Hospital of Central South University, Hunan Provincial People’s Hospital, Hunan cancer hospital and Central hospital of Xiangtan, using plasma cell-free DNA (cfDNA) samples. Baseline patients and disease characteristics were extracted from institutional electronic health records and included age at diagnosis, Gleason score, site of metastatic disease, PSA level at sequencing, the median time from ADT initiation to CRPC, and treatments for CRPC before and after sequencing. Patients were divided into three subgroups based on the alteration status of the seven known MMR genes (MLH1, MLH3, MSH2, MSH6, PMS1, PMS2, and EPCAM). These three alteration states were pathogenic MMR alterations, MMR alterations of unknown significance, and wild-type MMR genes.

### Definition of Mutation Status

All loss-of-function alterations of MMR genes were coded as pathogenic, including deletion, nonsense mutations, frameshift, and splice site alterations. Missense mutations were considered to be of unknown significance unless specifically designated as pathogenic in the clinVar database.

### DNA Sequencing and Bioinformatics

cfDNA was extracted using the QIAamp Circulating Nucleic Acid Kit (Qiagen) according to the manufacturer’s instructions. The NimbleGen SeqCap EZ Choice capture panel and the IDT capture panel were used to capture the coding regions of genes including MLH1, MLH3, MSH2, MSH6, PMS1, PMS2, and EPCAM. Final libraries were sequenced on the Illumina NextSeq 500 (PE75) or NovaSeq 6000 (PE150).

Somatic mutations and germline mutations were called by Mutect2 and GATK’s Haplotype Caller (17) with a paired workflow or GATK (17), respectively. Variants were annotated by ANNOVAR (18). Germline variants on WBC samples were first filtered with a threshold of minimum coverage of 50× and an allele frequency of over 30%. Variants not on coding regions and synonymous mutations were filtered out. Further, variants with over 0.1% population minor allele frequency as annotated by the ExAC database were considered less functional and ignored in the downstream analysis. Somatic mutations from cfDNA samples were filtered with the following rules: (1) 10 allele reads support; (2) 1% allele frequency; (3) supporting reads should be below four in the WBC control; (4) mutation frequency should be five times higher than in the WBC control; (5) mutations should not occur more than two times in the PoN; and (6) no significant strand bias (GATK parameter FS > 60 for SNPs and FS > 200 for indels).

### Outcome Measures

Clinical outcome measures were defined using the PCWG3 criteria (19). Progression-free survival (PFS) was defined as PSA progression (as the time interval to developing a greater than or equal to 25% increase in the PSA level from baseline or nadir (and by ≥2 ng/ml) that required confirmation more than or equal to 3 weeks later), radiographic progression, and/or symptomatic progression.

### Statistical Analysis

All statistical analysis was conducted in the R programming language. Baseline characteristics were compared by Fisher’s exact test. The Kaplan–Meier method was used to estimate the PFS of different subgroups, and differences between groups were identified using the log-rank test in the survival package (v.2.44.1.1). A value of *P* < 0.05 was considered to be statistically significant.

## Results

### Patient Cohort and Clinical Characteristics

A total of 109 mCRPC patients were enrolled for this study. Patients were divided into three subgroups on the basis of MMR alteration status: pathogenic MMR alterations (*n* = 7), MMR alterations of unknown significance (*n* = 43), and wild-type MMR genes (*n* = 59). The baseline characteristics of the three subgroups are shown in Table 1. For the seven patients with pathogenic MMR alterations, the median age at diagnosis was 63.5, 42.9% had a Gleason score ≥8, 100% had bone metastasis, 42.9% were treatment naive, 57.1% had received abiraterone treatment (none of the patients received abiraterone after enzalutamide in the present cohort), 14.3% had received docetaxel treatment, and the median time of androgen deprivation therapy (ADT) (all the patients included in the study received standard ADT without concurrent docetaxel, abiraterone, or enzalutamide) initiation to CRPC diagnosis was 24 months (Q1–Q3: 17.6–33.0 months). The median age at diagnosis of the 43 patients with MMR alterations of unknown significance was 66, 62.8% had a Gleason score ≥8, 34.9% were treatment naive, 53.5% had received abiraterone, 30.2% had received docetaxel, and the median time of standard ADT initiation to CRPC was 19.1 months (Q1–Q3: 10–30 months). Fifty-nine patients with wild-type MMR genes were included for comparison. The median age of these patients at diagnosis was 66, 65.8% had a Gleason score ≥8, 98.3% had bone metastasis, 67.8% were treatment naive, 22% had received abiraterone treatment, 10.2% had received docetaxel treatment, and the median time of standard ADT initiation to CRPC was 20 months (Q1–Q3: 12.2–32 8 months).

**TABLE 1:** Baseline characteristics of patients.

Variable	Pathogenic MMR alterations (*n* = 7)	MMR alterations of unknown significance (*n* = 43)	Wild-type MMR (*n* = 59)
**Age at diagnosis, y**			
Median (Q1–Q3)	63.5 (61.3–68.8)	66 (61–69)	66 (63–71)
Unknown	1		2
**Gleason Score at diagnosis, N (%)**			
≤7	3 (42.9)	12 (27.9)	21 (31.6)
≥8	3 (42.9)	27 (62.8)	36 (65.8)
Unknown	1 (14.3)	4 (9.3)	2 (2.6)
**Site of metastatic, N (%)**			
Lymph node	6 (85.7)	41 (95.3)	59 (100)
Bone	7 (100)	41 (95.3)	58 (98.3)
Visceral	1 (14.3)	3 (7)	1 (1.7)
**PSA at sequencing, ng/ml (%)**			
0∼10	0	10 (23.3)	8 (13.6)
10∼20	2 (28.6)	2 (4.7)	14 (23.7)
20∼100	2 (28.6)	13 (30.2)	16 (27.1)
>100	3 (42.9)	17 (39.5)	19 (32.2)
Unknown		1 (2.3)	2 (3.4)
**Median time from ADT initiation to CRPC, month**			
Median (Q1–Q3)	24 (17.6–33)	19.1 (10–30)	20 (12.2–32.8)
Unknown	1	2	6
**Prior treatment for CRPC before sequencing**			
Treatment naive	3 (42.9)	15 (34.9)	40 (67.8)
Use of abiraterone, N (%)	4 (57.1)	23 (53.5)	13 (22)
Use of docetaxel, N (%)	1 (14.3)	13 (30.2)	6 (10.2)
**Posttreatment for CRPC after sequencing**			
Use of abiraterone, N (%)	3 (42.9)	7 (16.3)	25 (42.4)
Use of docetaxel, N (%)	1 (14.3)	10 (23.3)	34 (57.6)
Other treatments	3 (42.9)	26 (60.5)	0

*MMR, mismatch repair; ADT, androgen deprivation therapy; CRPC, castration-resistant prostate cancer.*

### Genomic Characteristics

Among the seven patients with pathogenic MMR alterations, three harbored germline mutations, while the other four had somatic events. The mutations were gEPCAM (p.R173fs), MSH6 (p.F1088fs), gPMS1 (c.2539delG), gMLH3 (p.D206fs), MSH6 (p.E744fs), EPCAM (c.77–2A > T), and MLH1 (p.A239fs). The genomic alterations are listed in Table 2. Homologous recombination deficiency (HRD) defects, including alteration by mutation or copy number loss, were identified in three of the seven pathogenic MMR mutation cases, all of which had two alterations in either BRCA2, ATM, PALB2, or CDK12. Notably, the AR gene showed hotspot missense mutations or copy number amplifications in all seven patients, and two patients carried two different AR mutations. The AR alteration prevalence is significantly higher in patients carrying MMR alterations, pathogenic MMR alterations, or MMR alterations of unknown significance than in patients with wild-type MMR (24/50 and 10/59, *P* = 7.8 × 10^–4^; 7/7 vs. 10/59, *P* = 2.5 × 10^–5^; 17/43 vs. 10/59, *P* = 0.013). Two patients harbored a TP53 stop-gain mutation or an RB1 copy number loss.

**TABLE 2:** List of pathogenic alteration of patients with pathogenic MMR mutations.

Patient No	MMR	HRD	AR	Other
1	gEPCAM (p.R173fs)		AR gain	
2	MSH6 (p.F1088fs)		AR (p.T878A) AR (p.V716M)	TP53 (p.R306X)
3	gPMS1 (c.2539delG)		AR (p.T878A)	
4	gMLH3 (p.D206fs)	ATM (c.185+ 1G > C) BRCA2 loss	AR gain	RB1 loss
5	MSH6 (p.E744fs)		AR (p.H875Y)	
6	EPCAM (c.77–2A > T)	PALB2 (p.P212fs) BRCA2 (p.F663fs)	AR gain	
7	MLH1 (p.A239fs)	CDK12 (p.D995fs) CDK12 (p.T1002fs)	AR (p.L702H) AR (p.T878A)	

*g, germline; HRD, homologous recombination deficiency.*

### Association Between MMR Status and Clinical Outcomes

**FIGURE 1:**
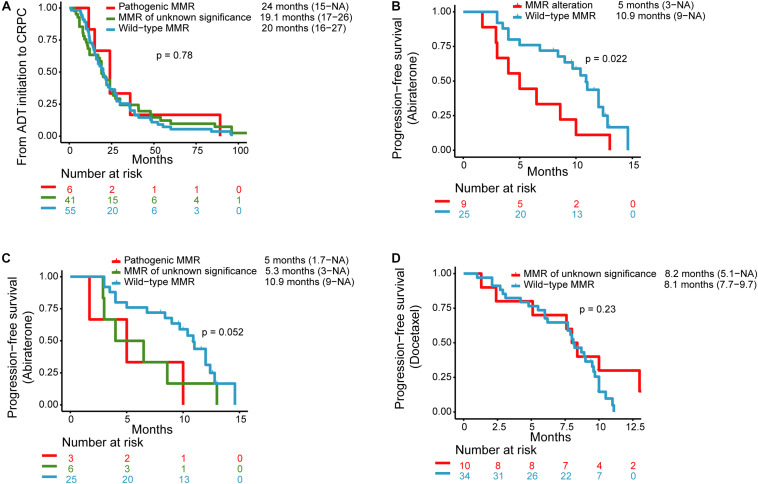
Kaplan–Meier curves for **(A)** progression-free survival (PFS) for androgen deprivation therapy (ADT) initiation to castration-resistant prostate cancer (CRPC) in patients with pathogenic MMR alterations, MMR alterations of unknown significance, and wild-type MMR. **(B)** PFS for abiraterone therapy in patients with MMR alterations and wild-type MMR. **(C)** PFS for abiraterone therapy in patients with pathogenic MMR alterations, MMR alterations of unknown significance, and wild-type MMR. **(D)** PFS for docetaxel therapy in patients with MMR alterations of unknown significance and wild-type MMR.

No significant difference was noted among the three subgroups with regard to the time from ADT initiation to develop CRPC. The median time from ADT initiation to CRPC diagnosis in patients with pathogenic, unknown significance, or wild-type MMR alterations was 24 months (range: 15–NA months), 19.1 months (range: 17–26 months), and 20 months (range: 16–27 months), respectively (*P* = 0.78) (Figure 1A).

Nine patients with MMR alterations (*n* = 3 for pathogenic MMR alterations, *n* = 6 for MMR alterations of unknown significance) and 25 patients with wild-type MMR received abiraterone and had clinical data available for PFS analysis. The median PFS for abiraterone in the MMR alterations group (*n* = 9) and the wild-type MMR (*n* = 25) were 5 months (range: 3 months–NA) and 10.9 months (range: 9 months–NA), respectively (*P* = 0.022) (Figure 1B). The median PFS for those with pathogenic MMR alterations (*n* = 3) and MMR alterations of unknown significance (*n* = 6) was 5 months (range: 1.7 months–NA) and 5.3 months (range: 3 months–NA), respectively (Figure 1C).

One patient with pathogenic MMR alterations received docetaxel after the genetic test, but without available clinical data. Ten patients with MMR alterations of unknown significance and 34 patients with wild-type MMR received docetaxel chemotherapy and had median PFS of 8.1 months (range: 5.1 months–NA) and 8.2 months (range: 7.7–9.7 months), respectively (Figure 1D).

## Discussion

Here we describe the clinical characteristics of patients with MMR alterations and examine the association between MMR alterations and outcomes of patients treated with standard systemic therapies. We observed that patients with any MMR alterations had inferior responses to abiraterone therapy. To our knowledge, this is the first study to evaluate the clinical features and treatment outcomes of dMMR mCRPC in Asian populations.

Mismatch repair gene mutations are rarely seen in prostate cancers. A whole-exome analysis of 150 metastatic site biopsies from castration-resistant patients showed that 2.0% had aberrations in MSH2, and 0.7% had aberrations in MLH1 (20). A recent study identified 3.7% of metastatic prostate cancers with deleterious alterations in MSH2, MSH6, or MLH1 from cfDNA samples (21). Previous studies reported that dMMR prostate cancer is an aggressive disease with a higher Gleason score, lower differentiation, and a higher rate of distant metastasis (16, 22, 23). In our study, three of six pathogenic MMR cases had a Gleason score ≥8. We did not observe significant differences in the rate of a high Gleason score among the three subgroups, which may be due to the small number of samples.

Abida et al. reported that 31 patients with MSI-H/dMMR prostate adenocarcinoma responded poorly to ADT therapy, and that the median time to castration resistance was 8.6 months (15). However, Antonarakis et al. reported that 13 MMR-deficient patients demonstrated high sensitivity to standard ADT therapies, with a median PFS of 66 months (16). In our study, the median PFS from ADT initialization to CRPC of the three subgroups, which included pathogenic MMR alterations, MMR alterations of unknown significance, and wild-type MMR, was 24, 19.1, and 20 months, respectively (*P* = 0.78). This suggests that MMR alterations do not have statistically significant effects on ADT therapies. The relationship between MMR alterations and response to ADT therapy may need larger cohort studies to be accurately determined.

The association between MMR alterations and response to abiraterone is still controversial. It has been reported that sensitivity to first-line abiraterone or enzalutamide was high among six dMMR patients, of whom 83% achieved a >50% PSA response, and among whom the median PFS was 26 months (16). In a retrospective study, MSI-H/dMMR mCRPC patients responded poorly to first-line treatment with abiraterone acetate or enzalutamide, with a median PFS of 9.9 months (15). Ritch et al. reported that the median duration for first-line androgen receptor pathway inhibitor therapy was only 3.9 months in 11 dMMR patients (21). Our findings are highly consistent with these results. The median PFS of those with MMR alterations and pathogenic MMR alterations was 5 months, which is much shorter than the wild-type MMR subgroup, which had a median PFS of 10.9 months. These results may be explained by the high concurrence of AR variations with MMR alterations. We observed that in the MMR alterations and pathogenic MMR alterations, there was a high concomitance between AR copy number amplification or AR ligand-binding domain mutations, compared with only 10/59 of the wild-type MMR subgroup (24/50 vs. 10/59, *P* = 7.8 × 10^–4^; 7/7 vs. 10/59, *P* = 2.5 × 10^–5^). These aberrations of the AR gene can confer resistance to abiraterone or enzalutamide (24, 25). It is noted that patients with MMR alterations of unknown significance (*n* = 6) also displayed resistance to abiraterone with a median PFS of 5.3 months. Though AR alteration prevalence is still significantly higher in patients with MMR alterations of unknown significance compared with wild-type MMR (17/43 vs. 10/59, *P* = 0.013), only one patient harbored AR alteration in those patients (*n* = 6) who received abiraterone. So, whether all the patients with MMR alterations respond poorly to novel hormone therapy, and the potential mechanism, remains unclear and requires verification with a larger prospective study.

Taxane-based chemotherapy is an alternative for metastatic castration-resistant prostate cancers. Our findings demonstrated that there was no difference in response to docetaxel chemotherapy between patients with MMR alterations of unknown significance and wild-type MMR patients.

One limitation of this study is that we were unable to control for potential baseline clinical discrepancies due to the retrospective nature of the analysis. Another limitation is the relatively small sample size used for the Kaplan–Meier curve analysis, so the results of those analyses should be interpreted with caution.

Overall, these results showed that dMMR mCRPC patients had equivalent responses to both standard ADT and taxane-based chemotherapy treatments compared with wild-type MMR patients. Patients with both pathogenic alterations and alterations of unknown significance to MMR genes had poorer responses to first-line or second-line abiraterone therapy. Future prospective studies with larger cohorts will provide more insight about the predictive value of MMR gene mutation in prostate cancer.

## Data Availability Statement

The datasets presented in this article are not readily available because of restrictions by national legislation/guidelines, specifically the Administrative Regulations of the People’s Republic of China on Human Genetic Resources. Requests to access the datasets should be directed to the corresponding author.

## Ethics Statement

This study was approved by the ethics committee of The Second Xiangya Hospital of Central South University. The patients provided their written informed consent to participate in this study.

## Author Contributions

SY, HW, RC, and LY participated in the conception and design of the study. SY, HW, KH, MP, YW, YL, SJ, and JL conducted the data collection and statistical analysis. SY and HW were responsible for the accuracy of the data analysis and drafted the manuscript. All authors approved the final manuscript.

## Conflict of Interest

The authors declare that the research was conducted in the absence of any commercial or financial relationships that could be construed as a potential conflict of interest.
